# Retinoic Acid Regulates Endothelial β-catenin Expression and Pericyte Numbers in the Developing Brain Vasculature

**DOI:** 10.3389/fncel.2018.00476

**Published:** 2018-12-05

**Authors:** Stephanie Bonney, Brenna J. C. Dennison, Megan Wendlandt, Julie A. Siegenthaler

**Affiliations:** ^1^Section of Developmental Biology, Department of Pediatrics, University of Colorado, Aurora, CO, United States; ^2^Cell Biology, Stem Cells and Development Graduate Program, University of Colorado, Aurora, CO, United States

**Keywords:** β-catenin, brain vasculature, pericytes, retinoic acid, Sox17, WNT signaling

## Abstract

The acquisition of brain vascular properties, like tight junctions and pericytes, to form the blood-brain barrier (BBB) is crucial for a properly functioning central nervous system (CNS). Endothelial WNT signaling is a known driver of brain vascular development and BBB properties, however, it is unclear how endothelial WNT signaling is regulated. We recently showed that mouse embryos with disruptions in endothelial retinoic acid (RA) signaling have ectopic WNT signaling in the brain vasculature. Using immunohistochemistical analysis, we show that increased vascular WNT signaling in RA mutants (*Pdgfbi*^cre^; *dnRAR403-flox* and *Rdh10* mutants) is associated with elevated expression of the WNT transcriptional effector, β-catenin, in the brain endothelium. *In vitro* immunocytochemistry and proximity ligation studies in brain endothelial cells reveal that RA, through its receptor RARα, regulates β-catenin expression in brain endothelial cells via transcriptional suppression and phosphorylation events that targets β-catenin for proteasomal degradation, the latter dependent on PKCα. We find that one function of RA in regulating vascular WNT signaling is to modulate the pericyte numbers in the developing brain vasculature. RA-mediated regulation of vascular WNT signaling could be needed to prevent over-recruitment of pericytes that might impair endothelial-pericyte interactions crucial for vascular stability.

## Introduction

As the central nervous system (CNS) develops, the brain vasculature grows and matures to support proper neural growth and function. The growing brain vasculature made up of endothelial cells quickly obtain unique brain vascular features to support neural growth and function. Important features acquired by the developing brain vasculature include tight junction assembly, expression of transporters, maintaining low-rates of transcytosis and immune invasion, and establishing a high density of pericytes that cover the vasculature. Through these features, the brain blood vessels form the blood-brain barrier (BBB) to limit the infiltration of harmful substances and provide a favorable ionic environment that supports neuronal activity (Hawkins and Davis, 2005; Obermeier et al., 2013; Bauer et al., 2014; Engelhardt and Liebner, 2014). Recent studies have made strides in our understanding of signals that promote acquisition of these brain vascular features by the developing brain vasculature, however, it is unclear how these signals are regulated to ensure proper acquisition of these properties.

WNT signaling is a major pathway important for growth of the brain vasculature and regulates blood vessel features in the brain, like BBB properties. WNT signaling occurs in brain endothelial cells when neuroepithelial-derived WNT ligands (WNT7a/7b) bind the Frizzled and LRP receptors (LRP5/6) expressed by the brain endothelial cells. This results in the sequestration and silencing of the β-catenin destruction complex (Axin/APC/GSK), consequently halting the proteasomal degradation of β-catenin. β-catenin is then able to translocate to the nucleus and promote the expression of WNT target genes through interactions with TCF/Lef1 (Obermeier et al., 2013). Along with growth of the brain vasculature, endothelial WNT signaling appears to be important in regulating the acquisition of two main brain vascular features through: (1) The expression of Claudin-5 (Cldn5), a tight junction protein that plays a crucial role in sealing the endothelium and restricting movement of molecules from the blood supply into the neural tissue (Zhou et al., 2014). (2) The recruitment of pericytes to the brain vasculature that support BBB function (Reis et al., 2012). Although, endothelial WNT signaling is important for the development of the brain vasculature and acquisition of these brain vascular properties, tight regulation of the WNT signaling pathway appears to be important for vascular stability.

Recently we showed that retinoic acid (RA) functions to regulate WNT signaling and WNT-driven vascular development through two mechanisms: RA balances vascular WNT signaling by (1) acting as a positive regulator of WNT-mediated vascular growth and (2) inhibiting endothelial WNT signaling in a cell autonomous manner. Positive regulation of WNT-mediated vascular growth was attributed to RA-mediated suppression of WNT signaling antagonists (Bonney et al., 2016). However, it is currently unclear how endothelial RA signaling acts to inhibit WNT signaling. The aims of these studies were twofold: (1) to identify the underlying mechanism of how RA cell autonomously inhibits endothelial WNT signaling during brain vascular development. (2) To determine whether RA and WNT signaling work in concert to control the acquisition of WNT-mediated brain vascular properties.

## Materials and Methods

### Animals

Mice used for experiments here were housed in specific-pathogen-free facilities approved by AALAC and were handled in accordance with protocols approved by the University of Colorado Anschutz Medical Campus IACUC committee. The following mouse lines were used in this study: *PdgfbiCre* (Claxton et al., 2008), *dnRAR403-flox* (Rosselot et al., 2010), *Ctnnb1-flox* (*Ctnnb1*^LOF^; Brault et al., 2001), *Ctnnb1-exon3-flox* (*Ctnnb1*^GOF^; Messerschmidt et al., 2016), *Cdh5Cre*^ERT2^ (obtained from Ralf Adams), *Sox17-flox* (Spence et al., 2009), and *Ai14-flox* (Jackson Laboratories, Bar Harbor, ME, United States). The *Rdh10* ENU point mutation mice were obtained from Andy Peterson at Genentech (Ashique et al., 2012). To activate Cre-mediated recombinase activity, Tamoxifen (Sigma, St. Louis, MO, United States) was dissolved in corn oil (Sigma, St. Louis, MO, United States; 20 mg/ml) and 100 mμl was injected intra-peritoneal into pregnant females at E9.5 and E10.5 to generate *PdgfbiCre*; *Ctnnb1*^GOF^, *PdgfbiCre*; *dnRAR403-flox*, and *Cdh5-CreER*^T2^;*Sox17-flox* mutant animals. For generation of *PdgfbiCre*; *Ctnnb1*^LOF^ mutants, tamoxifen was administered to pregnant females on E11.5 and E12.5. For proximity ligation assays, *Cdh5cre*^ERT2/+^; *Ai14*^fl/+^ pups were used following intra-peritoneal injections with 50 μL Tamoxifen (1 μg/mL) at P0 and P1 to express Tdtomato within the vasculature. At P10, mice were anesthetized and transcardiac perfusions were performed with PBS to eliminate background from red blood cells and serum, followed by perfusions and fixation with 4% paraformaldehyde. The skull was removed, the brain was dissected out and then cryoprotected with 20% sucrose in PBS and subsequently frozen in OCT for immunohistochemical processing prior to proximity ligation assays.

### Immunohistochemistry

Fetuses (E13.5–E18.5) were collected and whole heads or brains were fixed overnight in 4% paraformaldehyde. All tissues were cryoprotected with 20% sucrose in PBS and subsequently frozen in OCT. Tissue was cryosectioned in 12 μm increments. Immunohistochemistry was performed on tissue sections as described previously (Zarbalis et al., 2007; Siegenthaler et al., 2009) using the following antibodies: mouse anti-β-catenin 1:100 (Cell signaling, Danvers, MA, United States), rabbit anti-Claudin-5 1:100 (Abcam, Cambridge, MA, United States), mouse anti-CoupTFII 1:100 (R&D Systems, Minneapolis, MN, United States), rabbit anti-Pdgfrβ 1:100 (Cell signaling, Danvers, MA, United States) and rabbit anti-Fibrinogen 1:500 (Abcam, Cambridge, MA, United States). Following incubation with primary antibody(s), sections were incubated with appropriate Alexafluor-conjugated secondary antibodies (Invitrogen, Carlsbad, CA, United States), Alexafluor 633-conjugated isolectin-B4 (Ib4; Invitrogen, Carlsbad, CA, United States), and DAPI (Invitrogen, Carlsbad, CA, United States). Immunofluorescent (IF) images were captured using a Nikon (Melville, NY, United States) i80 research microscope with Cool-Snap CCD-cooled camera or Zeiss (Thornwood, NY, United States) 780 LSM confocal microscope. Laser power and gain settings were always the same between control and mutant samples to accurately analyze expression of protein of interest.

### Image Analysis

Vascular β-catenin expression was determined in E13.5 *Wild-type* (*Rdh10*^+/+^ and *Rdh10*^+/-^; *n* = 6 animals) and *Rdh10* mutants (*Rdh10*^-/-^; *n* = 5 animals). Similarly, vascular β-catenin and Claudin-5 expression was determined in E18.5 *dnRAR*^fl/fl^ (*n* = 6 animals), and *Pdgfbi*^cre/+^; *dnRAR403*^fl/fl^ animals (*n* = 5 animals). To quantify the percent of vascular β-catenin or Claudin-5 expression within the vasculature the length (nm) of β-catenin and Claudin-5 expression was measured and normalized to the total length of Ib4^+^ blood vessels (nm) per immunofluorescent confocal image using Zen software. Pericyte coverage was determined in brains of: E13.5 *Wild-type* (*Rdh10*^+/+^ and *Rdh10*^+/-^; *n* = 6 animals) and *Rdh10* mutants (*Rdh10*^-/-^; *n* = 5 animals); E18.5 *dnRAR*^fl/fl^ (*n* = 6 animals), and *Pdgfbi*^cre/+^; *dnRAR403*^fl/fl^ (*n* = 5 animals); E14.5 *Ctnnb1*^GOF/+^ (*n* = 4 animals) and *Pdgfbi*^cre/+^; *Ctnnb1*^GOF/+^ (*n* = 4 animals); *Pdgfbi*^cre/+^; E14.5 *Ctnnb1*^LOF/+^ (*n* = 3 animals) and *Pdgfbi*^cre/+^; *Ctnnb1*^LOF/LOF^ (*n* = 3 animals); E14.5 *Sox17*^fl/fl^ (*n* = 6 animals) and *Cdh5*^CreERT2/+^; *Sox17*^fl/fl^ (*n* = 6 animals). To quantify pericyte coverage the number of Pdgfrβ/CoupTFII^+^ cells surrounding the Ib4^+^ vasculature was counted. Pdgfrβ localizes to the membrane of pericytes while CoupTFII labels pericyte nuclei (along with some neuronal nuclei and venous endothelial cells), thus a pericyte was counted if it was both Pdgfrβ and CoupTFII positive and surrounding the Ib4 labeled vasculature. The number of pericytes were quantified using this method and divided by the total length of Ib4^+^ blood vessels (μm) and then multiplied by 100 to achieve number of pericytes per 100 μm of blood vessels. All analysis was performed using Zen software on 3–5 20x images per brain/animal. Analysis on the *Pdgfbi*^cre/+^; *dnRAR403-flox* experiments were blinded. Analysis on the *PdgfbiCre*; *Ctnnb1*^GOF^ animals were not blinded. Due to obvious vascular defects, analysis on *PdgfbiCre*; *Ctnnb1*^LOF^, *Rdh10*, and *Cdh5-CreER*^T2^;*Sox17-flox* animals were not blinded.

### Whole Brain Transcriptional Analysis

Meninges were removed from the brains of *Cdh5*^creERT2^; *Sox17*^fl/fl^ (E14.5; *n* = 11 animals), and *Sox17*^fl/fl^ (E14.5; *n* = 6 animals). RNA was isolated from whole brains with Qiagen RNAeasy (Hilden, Germany). cDNA was then synthesized using iScript cDNA synthesis kit (BioRad, Hercules, CA, United States) and qRT-PCR was performed to analyze WNT signaling (*Axin2* and *Lef1*), *Pdgfb*, and *Pdgfr*β. *Actb* transcript levels were also assessed and used to normalize expression levels. Delta-delta Ct analysis was performed and fold change over control is reported. *Actb* forward: CTAGGCACCAGGGTGTGAT, *Actb* reverse: TGCCAGATCTTCTCCATGTC; *Axin2* forward: GTGCCGACCTCAAGTGCAA, *Axin2* reverse: GGTGGCCCGAAGAGTTTTG; *Lef1* forward: AGGGCGACTTAGCCGACAT, *Lef1* reverse: GGGCTTGTCTGACCACCTCAT; *Pdgfb* forward: GGAGTCGAGTTGGAAAGCTCA, *Pdgfb* reverse: ACCAGGAAGTTGGCGTTGGT; *Pdgfr*β forward: CTGTGAATGCCGTGCAGACT, *Pdgfr*β reverse: TGGAAGTTCACCACATCATTGC.

### bEnd.3 Cell Line and Treatments

The mouse brain endothelioma cell line (bEnd.3) was obtained from ATCC (Manassas, VA, United States; cat# CRL-2299). All experiments were performed on cells from passages 2–15 and cells were grown in Dulbecco’s minimal essential media with 4.5 g/L glucose, 1.5 g/L sodium bicarbonate, 4 μm L-glutamine (Invitrogen, Carlsbad, CA, United States), 10% fetal bovine serum (FBS) (Invitrogen, Carlsbad, CA, United States) and Penicillin (0.0637 g/L)-Streptomycin (0.1 g/L). Drugs and concentrations used are as followed: vehicle (DMSO; Sigma, St. Louis, MO, United States), 50 nM RA (all-trans RA; Sigma, St. Louis, MO, United States), 1 μM pan-Retinoic acid receptor inhibitor (AGN 194310; APExBIO, Boston, MA, United States), 1 μM Protein kinase C inhibitor (bisindoylmaleimide I; Tocris, Minneapolis, MN, United States), and 100 nM Proteasome inhibitor (MG132; Tocris, Minneapolis, MN, United States). Cells were allowed to grow to ∼80% confluency and serum starved overnight prior to treatments with RA, RARi, PKCi, and/or Protease inhibitor.

### mRNA Analysis in bEnd.3 Cells

*Ctnnb1* expression was analyzed in the bEnd.3 cells following 24hr vehicle or RA exposure +/- RARi. Cells were then lysed with RLT buffer, RNA was isolated, cDNA was generated, and *Ctnnb1* expression was assessed by qRT-PCR and normalized to *Actb* expression. Delta-delta Ct analysis was performed and fold change over vehicle control is reported. Each independent experiment (*n* = 3) was performed on 3 separate passages with at least 3 samples per treatment condition (technical replicates). *Ctnnb1* forward: GGTGGGCTGGTATCTCAGAA, *Ctnnb1* reverse: CAAGCAAGGCTAGGGTTTGA.

### Immunocytochemistry in bEnd.3 Cells

Immunocytochemistry (ICC) experiments were performed on bEnd.3 cells plated on collagen-coated chambered slides (Thermo Fisher Scientific, Waltham, MA, United States). For experiments analyzing phospho-β-catenin expression or PKC activity (p-PKC substrate) bEnd.3 cells were treated for 24 h with vehicle or RA +/- RARi or +/- PKCi. For experiments analyzing total β-catenin expression bEnd.3 cells were treated for 48 h with vehicle or RA +/- RARi, +/- PKCi, or +/- Proteasome-inh. Following treatments, cells then were fixed with 100% methanol for 10 min and incubated with mouse anti-β-catenin antibody (1:100; Cell Signaling, Danvers, MA, United States), rabbit anti-phospho-β-catenin (1:100; Cell signaling, Danvers, MA, United States), or rabbit anti-phospho-PKC substrate (1:100; Cell Signaling, Danvers, MA, United States) for 1 h at room temperature. Cells were then incubated the appropriate Alexa-Fluor secondary and immunofluorescent images were captured using a Zeiss (Thornwood, NY, United States) 780 LSM confocal microscope. For fluorescent intensity quantification, images were analyzed using Zen imaging software and β-catenin, p-b-catenin, or p-PKCsub fluorescent intensity was normalized to total number of DAPI^+^ cells per 20x image. Each independent experiment (*n* = 3) was performed on 3 separate passages with 3–5 images captured and analyzed per treatment condition. Due to laser power and gain settings needing to be consistent between vehicle and treated samples to accurately analyze expression of protein of interest, imaging and analysis was not blinded.

### Proximity Ligation Assays in bEnd.3 Cells and Tissue

Proximity ligation assays (PLAs) were performed on bEnd.3 cells plated on collagen-coated chambered slides (Thermo Fisher Scientific, Waltham, MA, United States) following 0, 2, 4, and 8 h of treatment with RA. Cells were fixed for 10mins with 100% methanol and PLA assays were performed according to Duolink PLA protocol specifics (Sigma, St. Louis, MO, United States). To detect potential protein-protein interactions, samples were co-incubated for 1hr at room temperature with the following antibodies: β-catenin-RARα: mouse anti-β-catenin (1:100; Cell Signaling, Danvers, MA, United States) and rabbit anti-RARα (1:100; Santa Cruz Biotechnology, Santa Cruz, CA, United States); β-catenin-PKCα: mouse anti-β-catenin (1:100; Cell Signaling, Danvers, MA, United States) and rabbit anti-PKCα (1:100; Cell Signaling, Danvers, MA, United States); RARα-PKCα: goat anti-RARα (1:100; Abcam, Cambridge, MA, United States) and rabbit anti-PKCα (1:100; Cell signaling, Danvers, MA, United States); β-catenin-VE-cadherin (positive control): mouse anti-β-catenin (1:100; Cell signaling, Danvers, MA, United States) and rabbit VE-cadherin (1:200; Abcam, Cambridge, MA, United States); ZO-1-RARα (negative control): mouse anti-ZO-1 (1:100; Thermo Fisher Scientific, Waltham, MA, United States) and rabbit anti-RARα (1:100; Santa Cruz Biotechnology, Santa Cruz, CA, United States). Following confocal imaging, analysis of PLA puncta was performed using ImageJ and threshold, particle analysis. Number of puncta were normalized to total number of DAPI^+^ bEnd.3 cells to get the number of puncta/cell. The number of ZO-1-RARα puncta/cell (on average ∼0.17 puncta/cell) were determined in all conditions and subtracted as background from all protein-protein interaction analyses. Because imaging settings (laser power and gain) had to be set to positive controls and positive PLA staining, experiments were not blinded. PLA experiments were also performed on perfused PFA-fixed brain sections (no antigen retrieval) from postnatal day 10 *Cdh5cre*^ERT2/+^; *Ai14*^fl/+^ to detect these protein-protein interactions within the Tdtomato-expressing vasculature, however, antibody incubations were performed overnight at 4°C. Immunofluorescent images were captured using a Zeiss (Thornwood, NY, United States) 780 LSM confocal microscope where laser power and gain settings were set the same between experimental, positive and negative control samples.

### Microvessel Isolation, Multi-Gene Transcriptional Profiling

Microvessels were isolated from E18.5 (*n* = 3 animals) *Pdgfbi*^cre/+^;*Ctnnb1*^LOF/LOF^ and *Pdgfbi*^cre^^/+^;*Ctnnb1*^LOF/+^ brains using PECAM/CD31-coated magnetic beads as previously described (Siegenthaler et al., 2013). RNA was isolated, cDNA was generated, and multigene transcriptional profiling, a form of quantitative RT-PCR, was used to determine the number of mRNA copies per cell normalized to 18S rRNA abundance (10^6^ 18S-rRNA copies/cell) (Shih and Smith, 2005). For each sample, mRNA copy numbers for *Lef1*, *Axin2*, *Sox17*, *Pdgfb*, and *Pdgfr*β were normalized to *CD144* copy number to correct for variability in microvessel isolation between brains. *Cd144* forward: CAACTTCACCCTCATAAACAACCAT, *Cd144* reverse: ACTTGGCATGCTCCCGATT; *Sox17* forward: GGCCGATGAACGCCTTTAT, *Sox17* reverse: AGCTCTGCGTTGTGCAGATCT.

### Sox17 Knock-Down Experiments and Chromatin-Immunoprecipitation in bEnd.3 Cells

For Sox17 knock-down experiments, cells were plated on 24-well plates and allowed to grow to ∼70% confluency prior to siRNA transfection with non-targeting control (Scrambled) and mouse Sox17-targeting siRNA (SMARTpool; Dharmacon, Lafayette, CO, United States). siRNA transfection was performed using Dharmefect 4 and according to Dharmacon siRNA transfection specifications. Expression levels of *Sox17* and *Pdgfb* from four independent experiments (*n* = 4) were analyzed via qRT-PCR after 48 h of transfection. Chromatin-immunoprecipitation (ChIP) assays for Sox17 were performed using the SimpleChIP Enzymatic Chromatin IP Kit (Cell Signaling, Danvers, MA, United States). Specifically the bEnd.3 cells were grown on 5-15cm until they reached ∼80–90% confluency and then fixed with 1.5% EM-grade formaldehyde for 20 min (Polysciences Inc., Warrington, PA, United States). After stopping the fixation process, the DNA was digested with 1,000 gel units of nuclease for 10 min at 37°C. The nuclei were then sonicated 5 × 10 s at 100% power and then incubated on ice for 30 min to ensure efficient lysis of the nuclei. ChIP was performed at 4°C overnight on 10 mg of DNA with 10 μg of antibodies against α-Sox17 (*n* = 4 independent experiments; R&D Systems, Minneapolis, MN, United States), α-HistoneH3 (*n* = 2 independent experiments; Cell Signaling, Danvers, MA, United States), and α-Rabbit IgG (*n* = 2 independent experiments; Cell Signaling, Danvers, MA, United States). Once eluted, the ChIP-DNA and input were incubated with proteinase K overnight at 65°C. After DNA purification, qRT-PCR was performed for 8 potential Sox17 binding sites up-stream and within the *Pdgfb* locus were probed for using SsoAdvanced Sybr Green (BioRad, Hercules, CA, United States). Potential consensus binding sites were identified using biogrid-lasagna which identified variations of the Sox17 binding sequence (A/TA/TGAA/TG) in the *Pdgfb* locus. The following primers against potential Sox17 binding sites were used: Binding site within -3778–3763 bps (AAACAGTT) forward: TTCCTCCCCGTATTGCTTTT, reverse: TGTTAGACCTCTGCTGGCTG; Binding site within -2883–2866 bps (CATACACG) forward: GGTGAGCCATCTCTTCATCC, reverse: GCCCGACTATAAAGCAGCAG; Binding site within -2532–2515 bps (TCCTTCAGG) forward: CTCCACCCCTCATGTCTGTT, reverse: CCCAATAAGGAGGCGTTTTT; Binding site within -2282–2265 bps (CATGCATA) forward: ATACCTGGTGGCTCACAACC, reverse: AGGTGTTTTGTCTGCGTGTG; Binding sites within -1646–1640 bps (CTCATTGGC and AACACTGTC) forward: AGGTGACTGGAAAACCTCCA, reverse: TCCCGATGCCTGTTTAGATG; Binding site within -1445–1436 bps (CCCACTGTC) forward: TCCCGATGCCTGTTTAGATG, reverse: CAGAGGATCGTGGGAAAATG; Binding site within +805-822bps (CATGAATCG) forward: GGAGCCCACCCTCCTC, reverse: AGCGATTCATGCCGACTC.

### Statistics

To detect statistically significant differences in mean values of control and mutant genotypes at one developmental time point (vascular β-catenin or Claudin-5 expression, pericyte density, qPCR analysis), Student t tests were used. For analysis that compared more than two groups (e.g., multiple treatment conditions, timepoints) we used a one-way analysis of variance (ANOVA) with Tukey’s *post hoc* analysis was used to detect statistically significant differences between treatment conditions or timepoints. *P*-values less than 0.05 were considered statistically significant in these studies with specific *p*-values reported on all graphs. The standard deviation (SD) is reported on all graphs.

## Results

### Retinoic Acid Regulates β-catenin Expression in Brain Endothelial Cells

**FIGURE 1:**
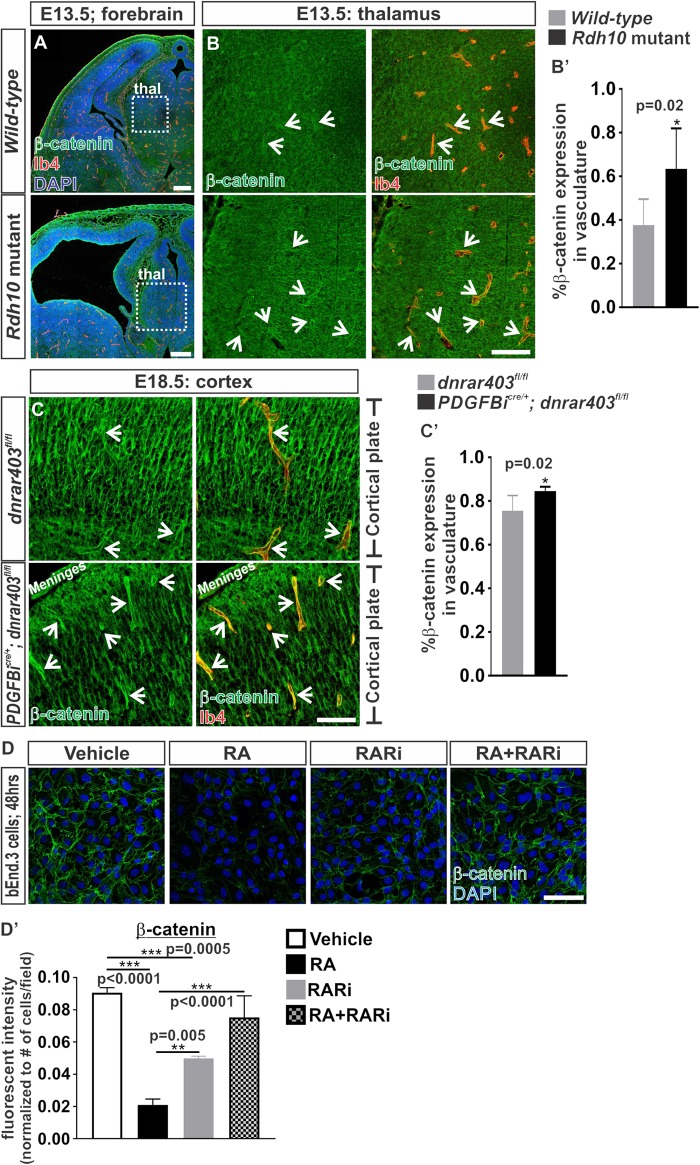
Retinoic acid regulates β-catenin expression in brain endothelial cells. **(A)** Immunofluorescent images of coronal forebrain sections from E13.5 *Wild-type* (*Rdh10*^+/+^ or *Rdh10*^+/-^) and *Rdh10* (*Rdh10*^-/-^) mutants stained for β-catenin (green), Isolectin-b4 (Ib4; red), and DAPI (blue). Scale bars are 200 μm. **(B,C)** Immunohistochemical images of **(B)** thalamic (thal) regions from E13.5 *Wild-type* (*Rdh10*^+/+^ or *Rdh10*^+/-^; inset from **A**) and *Rdh10* (*Rdh10*^-/-^; inset from **A**) mutants and **(C)** cortical plate from E18.5 *dnRAR403*^fl/fl^ and *Pdgfbi*^cre/+^; *dnRAR403*^fl/fl^ brains stained for β-catenin (green) and Ib4 (red). Arrows indicate positive β-catenin expression within the Ib4^+^ vasculature. Scale bars are 50 μm. **(B’,C’)** Quantification and analyses (Student’s *t*-test) for percent of β-catenin expression within the total Ib4^+^ labeled blood vessels from **(B’)** *Wild-type* (gray; *n* = 6 animals) and *Rdh10* mutant (black; *n* = 5 animals) and **(C’)** *dnRAR403*^fl/fl^ (gray; *n* = 6 animals) and *Pdgfbi*^cre/+^; *dnRAR403*^fl/fl^ (black; *n* = 5 animals). **(D)** Immunocytochemistry of β-catenin (green) expression in bEnd.3 cells following 48 h of vehicle, 50 nM RA, 1 μM RARi (AGN-194310), and RA + RARi. Scale bars are 50 μm. **(D’)** Quantification and analyses (ANOVA with Tukey’s *post hoc* analysis) on fluorescent intensity of β-catenin expression normalized to total number of DAPI+ bEnd.3 cells per field in vehicle (white), RA (black), RARi (gray), RA+RARi (black and gray) treated cells (*n* = 3 independent experiments).

Our previous work showed that RA cell autonomously regulates endothelial WNT signaling in the developing brain vasculature (Bonney et al., 2016). Other work in various cell types have suggested that RA is capable of reducing the expression of the WNT transcriptional effector, β-catenin (Lim et al., 2012; Zhu et al., 2015; Zito et al., 2017). Therefore, it is possible that RA regulates β-catenin expression to modulate endothelial WNT signaling. Thus we investigated the protein expression of β-catenin in the brain vasculature of mouse mutants that have an ENU point mutation in the RA synthesizing enzyme, *Rdh10*, resulting in an embryo-wide reduction of RA and RA signaling (Ashique et al., 2012). The RA-mediated inhibition of endothelial WNT signaling was observed within the non-neocortical vasculature of *Rdh10* mutants (Bonney et al., 2016). We therefore analyzed expression of β-catenin in the non-neocortical vasculature at E13.5 (this mutation in *Rdh10* results in embryonic lethality by E14.5). Surprisingly we found a significant increase in the expression of β-catenin within the non-neocortical (thalamus) vasculature of *Rdh10* mutants (Figures 1A,B,B’). We also observed a potential increased expression of neural β-catenin, potentially due to the embryo-wide reduction in RA synthesis and signaling (Figure 1B).

We next took an endothelial-specific approach by disrupting RA signaling within the developing vasculature. To do this we expressed a dominant-negative retinoic-acid receptor (RAR) allele, *dnRAR403*, in the developing vasculature using the endothelial-specific Cre recombinase *Pdgbi*^cre^ (*Pdgfb-CreER*^T2^) where expression of *dnRAR403* essentially silences endogenous RA-RAR signaling (Tsai et al., 1992; Damm et al., 1993; Lohnes et al., 1994). Expression of *dnRAR403* in the vasculature does not alter survival of the embryos and thus permits later analysis of a more mature brain vasculature than the *Rdh10* mutants. Like the non-neocortical vasculature of *Rdh10* mutants, we also showed previously that *Pdgfbi*^cre/+^; *dnRAR403*^fl/fl^ embryos have elevated endothelial WNT signaling in the developing neocortical brain vasculature (Bonney et al., 2016). Associated with this, we found a significant elevation in β-catenin expression in the neocortical brain vasculature of *Pdgfbi*^cre^; *dnRAR403*^fl/fl^ embryos when compared to *dnRAR403*^fl/fl^ controls (Figures 1C,C’). We next tested the sufficiency of RA to suppress β-catenin protein expression using bEnd.3 cells, a brain endothelioma cell line. Immunocytochemical analysis of β-catenin in bEnd.3 cells showed reduced expression after 48 h of 50 nM *all-trans* RA (hereafter referred to as RA) treatment, a concentration we previously showed inhibits WNT signaling (Figures 1D,D’). The RA-mediated suppression of β-catenin protein expression in the bEnd.3 cells was blocked with the addition of a pan RAR inhibitor, AGN 194310 (Figures 1D,D’). Of note, due to lack of WNT ligand exposure in these experiments, β-catenin localizes to the adheren junctions and not the nucleus. Together, this indicates that RA is sufficient to control β-catenin expression in brain endothelial cells and this depends on RAR activity.

**FIGURE 2:**
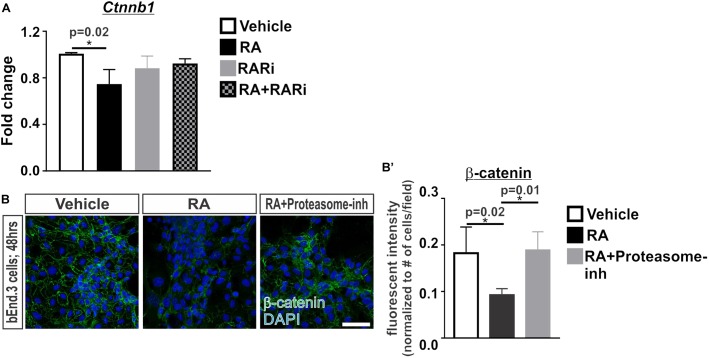
Retinoic acid regulates β-catenin expression via transcription and proteasomal degradation in brain endothelial cells. **(A)** Transcript analysis (ANOVA with Tukey’s *post hoc* analysis) of *Ctnnb1* expression in bEnd.3 cells following 24 h of vehicle (white), 50 nM RA (black), 1 μM RARi (AGN-194310; gray), and RA + RARi (black and gray) (*n* = 3 independent experiments). **(B)** Immunocytochemistry of β-catenin (green) and DAPI (blue) following 48 h of treatment with vehicle, RA, or RA + 100 nM Proteasomal inhibitor (Proteasome-inh; MG132) in the bEnd.3 cells. Scale bar is 50 μm. **(B’)** Quantification and analyses (ANOVA with Tukey’s *post hoc* analysis) of β-catenin fluorescent intensity normalized to the total number of DAPI+ bEnd.3 cells per field in vehicle (white), RA (black), RA + Proteasome-inh (gray) (*n* = 3 independent experiments).

We next investigated if RA is capable of regulating β-catenin transcriptionally or via proteasomal degradation. Transcriptional expression of *Ctnnb1* was significantly reduced following 24 h of RA exposure and this effect was blocked with the addition of the pan RAR inhibitor (Figure 2A). We further found that treatment with a proteasome inhibitor, MG-132, blocked the RA-mediated reduction in β-catenin protein expression (Figures 2B,B’). From this data we conclude that RA reduces β-catenin expression at the transcriptional level and via a mechanism that involves proteasomal degradation.

### Retinoic Acid Induces the Phosphorylation of β-catenin Through RAR and PKC Activity

**FIGURE 3:**
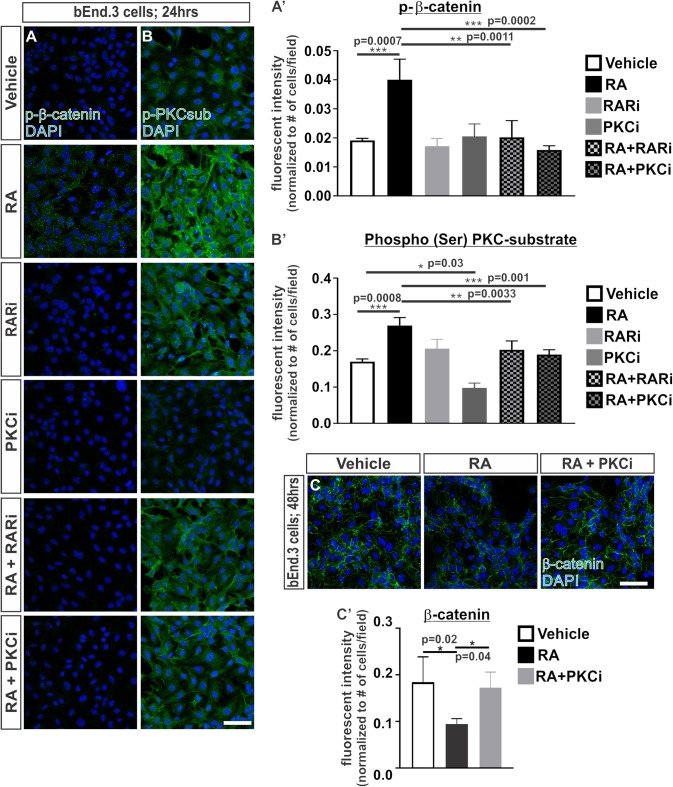
Retinoic acid induces the phosphorylation of β-catenin through RAR and PKC activity. **(A,B)** Immunocytochemistry (ICC) of **(A)** phospho-β-catenin at Ser33/Ser37/Thr41 (p-β-catenin; green) and **(B)** phospho-(Ser) PKC substrate (p-PKCsub; green) following 24 h of vehicle, 50 nM RA, 1 μM RARi (AGN-194310), 1 μM PKCi (bisindoylmaleimide I), RA+RARi, and RA+PKCi. (A and B’) Quantification and analyses (ANOVA with Tukey’s *post hoc* analysis) for fluorescent intensity of **(A’)** p-b-catenin and **(B’)** p-PKCsub normalized to the total number of DAPI+ bEnd.3 cells in vehicle (white), RA (black), RARi (light gray), PKCi (dark gray), RA+ RARi (light gray and black), and RA + PKCi (dark gray and black) (*n* = 3 independent experiments). **(C)** ICC of β-catenin (green) and DAPI (blue) following 48 h of treatment with vehicle, RA, or RA + PKCi in the bEnd.3 cells. **(C’)** Quantification and analyses (ANOVA with Tukey’s *post hoc* analysis) of β-catenin fluorescent intensity normalized to the total number of DAPI+ bEnd.3 cells per field in vehicle (white), RA (black), RA + PKCi (gray) (*n* = 3 independent experiments). Scale bars are 50 μm.

We undertook experiments to understand how RA may regulate the proteasomal-mediated degradation of β-catenin. We began by investigating whether RA promotes the phosphorylation of β-catenin at Ser33, Ser37 and Thr41, which generally marks β-catenin for proteasomal degradation (Verheyen and Gottardi, 2010). Following 24 h of RA exposure in the bEnd.3 cells, we saw an increase in the phosphorylation of β-catenin (Ser33/Ser37/Thr41) and this required RAR activity (Figures 3A,A’). RA has been shown to activate a number of kinases such as PKC (del Rincon et al., 2004; Miloso et al., 2004; Aggarwal et al., 2006) and PKC can phosphorylate β-catenin at Ser33/Ser37 and target it for degradation (Gwak et al., 2006). We next tested if the RA-mediated phosphorylation of β-catenin protein requires PKC by exposing bEnd.3 cells to a PKC inhibitor, bisindoylmaleimide I (BIM), with and without RA. Inhibiting PKC activity blocked the RA-mediated phosphorylation of β-catenin (Figures 3A,A’). Furthermore, RA treatment activated PKC activity as shown by an increase in the phosphorylation of PKC substrates (Figures 3B,B’). Treatment with BIM effectively reduced PKC phosphorylation activity with and without RA (Figures 3B,B’). Interestingly, treatment with the RAR inhibitor in the presence of RA also attenuated PKC phosphorylation activity (Figures 3B,B’) suggesting that RA requires RAR activity to activate PKC. The reduction in total β-catenin expression mediated by RA was blocked with addition of BIM (Figures 3C,C’). Together, this data suggests that RA activates PKC activity through RARs and this results in phosphorylation and the eventual degradation of β-catenin.

### Retinoic Acid Induces Interactions Between β-catenin With RARα and PKCα

**FIGURE 4:**
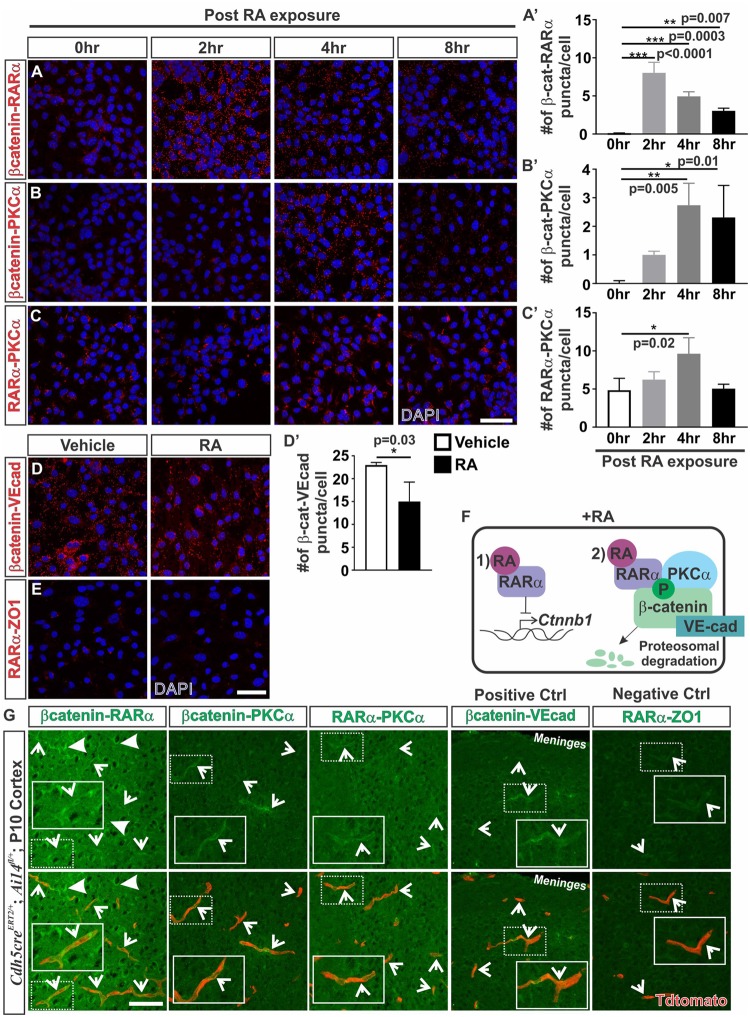
Retinoic acid induces interactions between β-catenin with RARα and PKCα. **(A–C)** Immunofluorescent (IF) images of proximity ligation assays (PLAs) for **(A)** β-catenin-RARα (red), **(B)** β-catenin-PKCα (red) and **(C)** RARα-PKCα (red) following 0, 2, 4, and 8 h of 50 nM RA exposure in bEnd.3 cells. **(A–C’)** Quantification and analyses (ANOVA with Tukey’s *post hoc* analysis) for number of **(A’)** β-catenin-RARα, **(B’)** β-catenin-PKCα, **(C’)** RARα-PKCα PLA puncta normalized to total number of DAPI^+^ bEnd.3 cells per field after 0 h (white), 2 h (light gray), 4 h (dark gray), and 8 h (black) of RA exposure (*n* = 3 independent experiments). **(D)** IF images of PLAs for β-catenin-VE-cadherin (VE-cad) following 24 hrs of RA exposure in bEnd.3 cells. **(D’)** Quantification and analyses (Student’s *t* test) for β-catenin-VEcad PLA puncta normalized to total number of DAPI^+^ bEnd.3 cells per field after 24 h of vehicle (white) or RA (black) (*n* = 3 independent experiments). **(E)** IF images of negative PLA control for RARα-ZO1 after 24 h of vehicle or RA treatment in bEnd.3 cells. **(F)** Model for how RA regulates β-catenin expression in brain endothelial cells: In the presence of RA, RARα is activated and regulates β-catenin via (1) transcriptional suppression of *Ctnnb1* gene expression and (2) phosphorylation events that target β-catenin for degradation through interactions with RARα and PKCα. **(G)** PLA IF images for β-catenin-RARα, β-catenin-PKCα, RARα-PKCα, β-catenin-VEcad (positive control), and RARα-ZO1 (negative control) in postnatal day 10 cortical tissue of *Cdh5cre*^ERT2/+^; *Ai14*^fl/+^ (Enlarged images of single vessels provided in insets). Arrows indicate positive PLA staining within the Tdtomato-expressing vasculature (and faint vascular staining in the negative control (RARα-ZO1). Scale bars are 50 μm.

Given the robust effect of RA on promoting β-catenin phosphorylation and protein degradation, we next investigated how RA may be functioning to phosphorylate β-catenin. RARα, which is highly expressed by the developing brain vasculature in mice (Bonney et al., 2016), has been shown to directly interact with β-catenin (Easwaran et al., 1999; Chanda et al., 2013). Possibly, RA treatment induces a complex formation between RARα, β-catenin, and PKC to target β-catenin for degradation via phosphorylation. To test this we performed proximity ligation assays (PLAs) which allowed us to assess if RARα, β-catenin and PKCα are within close proximity (∼40 nm) to one another, suggestive of complex formation. Following 2 h of RA exposure, we found a substantial increase in β-catenin-RARα PLA puncta, indicating that RARα and β-catenin were within close proximity to one another. The puncta were generally found within the cytoplasm and were sustained after 8 h of RA exposure (Figures 4A,A’). After 4 h of RA exposure we found a significant increase in β-catenin-PKCα puncta suggesting RA facilitates interactions between β-catenin and PKCα (Figures 4B,B’). Similar observations were noted in PLAs performed for RARα and PKCα after 4 h of RA exposure, however, it appears that RARα and PKCα may interact at baseline conditions, without the presence of RA (Figures 4C,C’). This suggests that under normal conditions RARα interacts with PKCα and in the presence of RA, RARα is then activated and binds to β-catenin where it then facilitates interactions between β-catenin and PKCα. These interactions could then allow for the phosphorylation events that target β-catenin for degradation. We found a significant reduction in PLA puncta for β-catenin-VE-cadherin following 24 h of RA exposure (Figures 4D,D’) suggesting that RA is capable of reducing β-catenin destined for or bound at the adheren junctions. Importantly, very few PLA puncta were observed in our negative control (RARα-ZO-1; Figure 4E). Collectively, our *in vitro* data suggests a model where in the presence of RA, RARα can (1) transcriptionally suppress *Ctnnb1* expression and (2) bind β-catenin and target it for degradation through phosphorylations via interactions with PKCα (Figure 4F).

We next looked for indication of these interactions in the brain vasculature. Due to high background from serum and red blood cell contamination in embryonic tissue, PLA experiments were performed on perfused postnatal (P10) *Cdh5creERT2/+*; *Ai14fl/+* tissue that had Tdtomato expression in the endothelium. We observed positive PLA staining for β-catenin-RARα within the Tdtomato-expressing vasculature (arrows; Figure 4G). We also noted puncta indicative of interactions between β-catenin and RARα in other cells within the brain (closed arrows; Figure 4G). Additionally, we found positive PLA interactions between β-catenin-PKCα, RARα-PKCα, and β-catenin-VE-cadherin (positive control) which appeared mostly within the Tdtomato-expressing vasculature (arrows; Figure 4G). A faint signal was detected in the negative control (RARα-ZO1) in the Tdtomato-expressing vasculature, however, this was at a much lower intensity than the other PLA interactions (arrows; Figure 4G). From these investigations, we conclude that RA regulates β-catenin expression in part through proteasomal degradation and this involves RA-mediated interactions between RARα, β-catenin and PKCα in the brain endothelium.

### Ectopic Vascular WNT-β-catenin Signaling in RA Mutants Result in Increased Pericytes Along the Developing Brain Vasculature

We next tested if RA inhibition of endothelial WNT signaling has any role in modulating WNT-driven brain vascular properties, like BBB formation. Ectopic WNT signaling in the developing vasculature of *Rdh10* and *Pdgfbi*^cre^; *dnRAR403*^fl/fl^ mutants (Bonney et al., 2016) could result in increased expression of the TJ protein and WNT-target, Cldn5, and impair BBB function. Our previous work addressing the role of RA in BBB development showed that *Rdh10* mutants do not have alterations in vascular protein expression of Cldn5 or overt leakage of fibrinogen by the brain vasculature (Bonney and Siegenthaler, 2017). Further, disrupting vascular RA signaling (*Pdgfbi*^cre^; *dnRAR403*^fl/fl^) did not result in up-regulation of Cldn5 in the vasculature (Supplementary Figures 1A,A’). Additionally, leakage of fibrinogen was not observed suggesting BBB function is normal in these animals (Supplementary Figure 1B). This suggests that endothelial RA does not function upstream of WNT-β-catenin signaling to modulate TJ protein expression.

**FIGURE 5:**
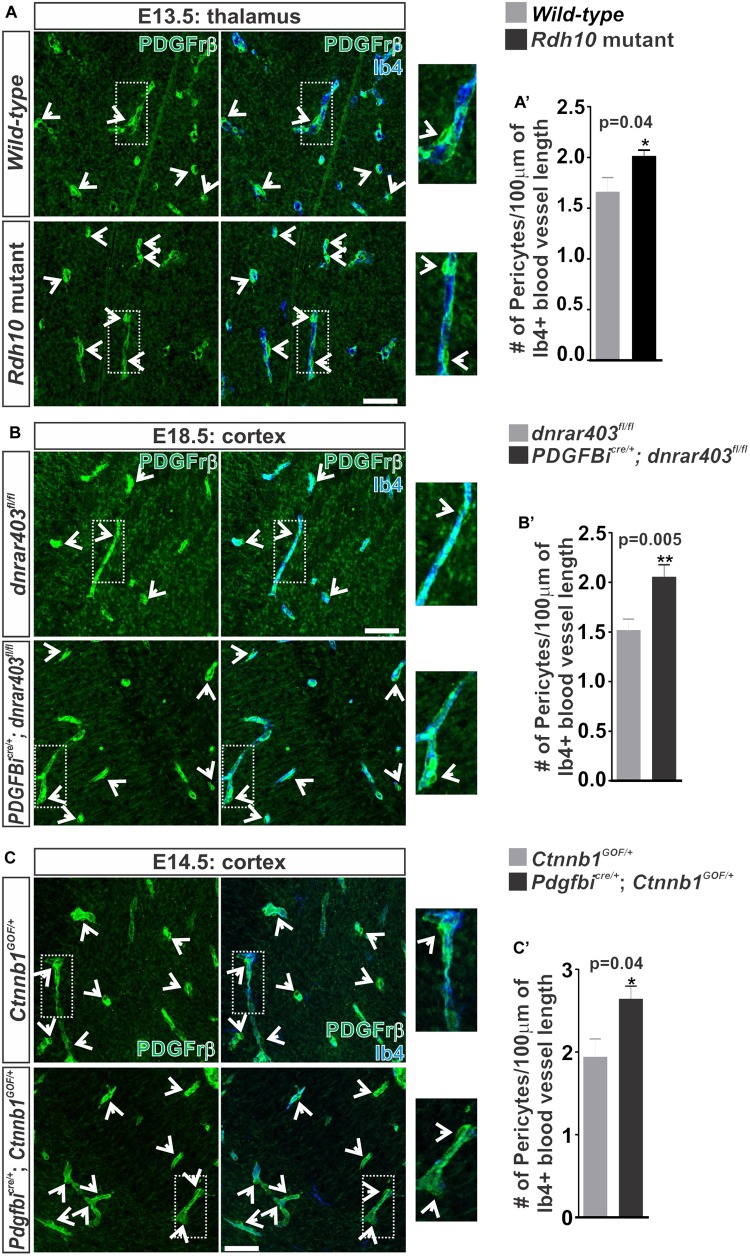
Ectopic vascular WNT signaling in RA mutants result in increased pericytes along the developing brain vasculature. **(A–C)** Immunohistochemical (IHC) images of the **(A)** thalamic regions from E13.5 *Wild-type* (*Rdh10*^+/+^ or *Rdh10*^+/-^) and *Rdh10* (*Rdh10*^-/-^), **(B)** cortical plate from E18.5 *dnRAR403*^fl/fl^ and *Pdgfbi*^cre/+^; *dnRAR403*^fl/fl^, and **(C)** cortical plate from E14.5 *Ctnnb1*^GOF/+^ and *Pdgfbi*^cre/+^; *Ctnnb1*^GOF/+^ brains stained for Pdgfrβ (green) and Isolectin-b4 (Ib4; blue). Enlarged images and arrows indicate Pdgfrβ^+^ pericytes surrounding the Ib4^+^ vasculature. **(A–C’)** Quantification and analyses (Student’s *t*-test) for the number of pericytes co-labeled for Pdgfrβ and CoupTFII (staining not shown)/100 μm of Ib4^+^ blood vessels from **(A’)** W*ild-type* (gray; *n* = 5 animals) and *Rdh10* mutants (black; *n* = 5 animals), **(B’)** *dnRAR403*^fl/fl^ (gray; *n* = 6 animals) and *Pdgfbi*^cre/+^; *dnRAR403*^fl/fl^ (black; *n* = 6 animals), and **(C’)** *Ctnnb1*^GOF/+^ (gray; *n* = 4 animals) and *Pdgfbi*^cre/+^; *Ctnnb1*^GOF/+^ (black; *n* = 4 animals). Scale bars are 50 μm.

In addition to TJ formation, WNT signaling may play a role in the recruitment of pericytes to the brain vasculature through regulation of pericyte mitogen and chemoattractant PDGF-B (Reis et al., 2012). We next tested if the increase in WNT-β-catenin signaling we observe in our RA signaling/synthesis mouse mutants (Bonney et al., 2016) increases pericyte numbers in developing brain vasculature. Indeed, we observed a significant increase in the number of Pdgfrβ^+^ pericytes covering the vasculature in *Rdh10* mutants (thalamus E13.5; Figures 5A,A’) and in *Pdgfbi*^cre/+^; *dnRAR403*^fl/fl^ embryos (cortex E17.5; Figures 5B,B’) when compared to controls. We next tested if ectopic WNT-β-catenin signaling is sufficient to increase pericyte numbers through a WNT gain-of-function approach by utilizing the *Ctnnb1-exon3-flox* (*Ctnnb1*^GOF^) mice crossed with *Pdgfbi*^cre^ mice. Over-activation of WNT-β-catenin signaling in the embryonic brain vasculature (E14.5; *Pdgfbi*^cre/+^; *Ctnnb1*^GOF/+^) significantly increased pericyte numbers (Figures 5C,C’). Our analysis suggests that ectopic vascular WNT signaling and β-catenin expression in the *Rdh10* and *Pdgfbi*^cre/+^^;^ *dnRAR403*^fl/fl^ mutants results in elevated pericyte numbers in the developing brain vasculature. Thus, appropriate brain pericyte recruitment may be an important function of RA in modulating endothelial WNT-β-catenin signaling.

### Vascular WNT Signaling and Sox17 Regulate Pericyte Numbers Along the Developing Brain Vasculature

**FIGURE 6:**
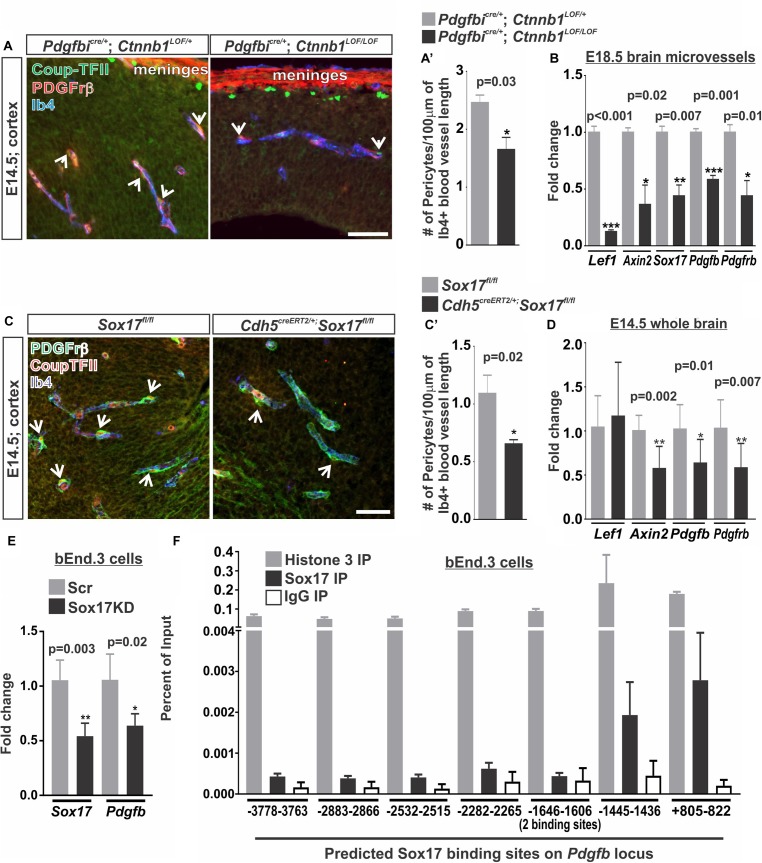
Vascular WNT signaling and Sox17 regulate pericyte numbers along the developing brain vasculature. **(A,C)** Immunohistochemical images of cortices from **(A)** E14.5 *Pdgfbi*^cre/+^; *Ctnnb1*^LOF/+^ and *Pdgfbi*^cre/+^; *Ctnnb1*^LOF/LOF^ and **(C)** E14.5 *Sox17*^fl/fl^ and *Cdh5*^CreERT2/+^; *Sox17*^fl/fl^ brains stained for Pdgfrβ (red), Coup-TFII (green) and Isolectin-b4 (Ib4; blue). Arrows indicate pericytes co-labeled for Pdgfrβ and Coup-TFII surrounding the Ib4^+^ vasculature. Scale bars are 50 μm. **(A’,C’)** Quantification and analyses (Student’s *t*-test) for the number of Pdgfrβ/CoupTFII co-labeled pericytes/100 μm of Ib4^+^ blood vessels from **(A’)** *Pdgfbi*^cre/+^; *Ctnnb1*^LOF/+^ (gray) and *Pdgfbi*^cre/+^; *Ctnnb1*^LOF/LOF^ (black) (*n* = 3 animals) and **(C’)** *Sox17*^fl/fl^ (gray) and *Cdh5*^CreERT2/+^; *Sox17*^fl/fl^ (black) (*n* = 6 animals) cortices. **(B)** Expressional analysis (Student’s *t*-test) of *Lef1*, *Axin2*, *Sox17*, *Pdgfb*, and *Pdgfr*β in brains microvessels isolated from *Pdgfbi*^cre/+^; *Ctnnb1*^LOF/+^ (gray) and *Pdgfbi*^cre/+^; *Ctnnb1*^LOF/LOF^ (black) embryos at E18.5 (*n* = 3 animals). **(D)** Expressional analysis (Student’s *t*-test) of *Lef1*, *Axin2*, *Sox17*, *Pdgfb*, and *Pdgfr*β in E14.5 whole brains of *Sox17*^fl/fl^ (*n* = 6 animals; gray) and *Cdh5*^CreERT2/+^; *Sox17*^fl/fl^ (*n* = 11 animals; black). **(E)** Expressional analysis (Student’s *t*-test) of *Sox17* and *Pdgfb* in bEnd.3 cells following 48 h of treatment with Sox17 or non-targeted control [scrambled (Scr)] siRNA (*n* = 4 independent experiments). **(F)** Chromatin Immunoprecipitation-qPCR for Histone H3 (*n* = 2 independent experiments; gray), Sox17 (*n* = 4 independent experiments; black) or rabbit-IgG (*n* = 2 independent experiments; white) in the bEnd.3 cells for 8 predicted Sox17 binding sites (AACAATGCAATTGTT) on the *Pdgfb* locus graphed to percent of input.

Our studies using the *Pdgfbi*^Cre/+^: *Ctnnb1*^GOF/+^ (Figure 5) and studies of pathological brain tumor vasculature demonstrate that increasing endothelial WNT-β-catenin signaling is sufficient to promote pericyte recruitment to the vasculature via PDGF-B (Reis et al., 2012). It is not known, however, if WNT-β-catenin is required for the high pericyte density in the developing brain vasculature. We tested this by conditionally knocking out β-catenin in the endothelium using the *Pdgfbi*^cre/+^; *Ctnnb1*^LOF/LOF^ animals. Compared to controls (*Pdgfbi*^cre/+^; *Ctnnb1*^LOF/+^), we found that *Pdgfbi*^cre/+^; *Ctnnb1*^LOF/LOF^ resulted in a significant reduction in number of pericytes along the vasculature (Figures 6A,A’). This correlated with a significant reduction in the expression of *Pdgfb* along with the WNT target genes (*Lef1*, *Axin2*, and *Sox17*) in brain microvessels isolated from *Pdgfbi*^cre/+^; *Ctnnb1*^LOF/LOF^ E14.5 embryos. We also detected a significant reduction in *Pdgfr*β since the Pdgfrβ-expressing pericytes are included in the microvessel isolations, further supporting a reduction in pericyte numbers (Figure 6B).

We next investigated how WNT-β-catenin signaling might regulate brain pericyte density. Direct regulation of the pericyte mitogen gene, *Pdgfb*, by the WNT transcriptional machinery, TCF/Lef1, seemed unlikely since we were unable to find TCF/Lef1 binding site motifs in or near the *Pdgfb* locus using the LASANGA transcription factor binding site search engine. Sox17, a transcription factor downstream of WNT signaling, has been shown to regulate mural cell coverage in the developing retinal vasculature (Corada et al., 2013). Our work and others have shown that WNT signaling is a major regulator of Sox17 during vascular development and disrupting endothelial RA signaling results in ectopic Sox17 expression in the brain vasculature (Ye et al., 2009; Corada et al., 2013; Bonney et al., 2016). We therefore assessed the role of Sox17 in regulating pericyte numbers by deleting *Sox17* in the vasculature of embryos (*Cdh5*^creERT2^; *Sox17*^fl/fl^). We found that deletion of *Sox17* in the endothelium (*Cdh5*^creERT2^; *Sox17*^fl/fl^ vs. *Sox17*^fl/fl^ embryos) resulted in reduced numbers of pericytes in the E14.5 brain vasculature (Figures 6C,C’). We also found a significant reduction in *Pdgfb*, *Pdgfr*β, and *Axin2* expression in whole brains of E14.5 *Cdh5*^creERT2^;*Sox17*^fl/fl^ embryos when compared to *Sox17*^fl/fl^ controls. *Lef1* expression was not significantly affected by loss of *Sox17* in the brain vasculature (Figure 6D). This suggests that Sox17 regulates pericyte numbers and *Pdgfb* expression during brain vascular development downstream of endothelial WNT signaling.

We next tested if Sox17 might be a direct transcriptional regulator of *Pdgfb* expression in brain endothelial cells. Knock-down of Sox17 in the bEnd.3 cells using siRNA resulted in a significant reduction in *Sox17* and *Pdgfb* expression when compared to Scrambled siRNA treated (Scr) control cultures (Figure 6E). We next analyzed the *Pdgfb* locus for Sox17 binding sites where we identified eight variations of the Sox17 consensus binding sites ranging from ^-^3778 bps upstream of the transcriptional start site to ^+^822 bps within the first exon. Chromatin Immunoprecipitation-qPCR assays (ChIP-qPCR) for Sox17 in the bEnd.3 cells showed an enrichment of Sox17 at ^-^1445–1436 bps and ^+^805–822 bps binding sites (Figure 6F). Positive control ChIP-qPCRs for histone H3 had a high percent of input while we found a low percent of input for the negative control (α-rat IgG) at all eight binding sites indicating the ChIP-qPCRs were successful and non-specific binding was minimal (Figure 6F). This data indicates that *Pdgfb* is potentially a direct transcriptional target of Sox17 in the brain endothelium.

## Discussion

**FIGURE 7:**
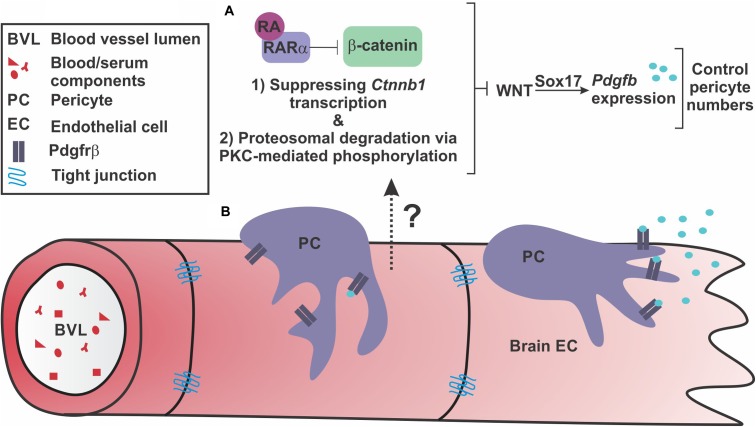
Retinoic acid regulates endothelial WNT signaling through β-catenin to appropriately modulate WNT-driven pericyte numbers along the developing brain vasculature. **(A)** Our investigations suggest that endothelial retinoic acid (RA) signaling regulates β-catenin through (1) transcriptional suppression of *Ctnnb1* and (2) phosphorylations that target β-catenin for degradation potentially through interactions with PKCα and RARα. Through these functions, RA controls pericyte numbers and PDGF-B expression through vascular WNT signaling and Sox17 expression. **(B)** This regulation may be important to appropriately establish Pdgfrβ-expressing pericytes along growing brain vasculature via Sox17-driven PDGF-B expression by the brain endothelium.

Our findings extend our previous work by identifying a mechanism by which RA regulates brain endothelial WNT-β-catenin signaling (Figure 7A). Further, our work suggests regulation of PDGF-B expression and brain pericytes via Sox17 as another potential facet of WNT-β-catenin regulation of BBB properties and a target of RA inhibition (Figure 7A).

Our work provides two mechanisms for how RA controls WNT-β-catenin signaling, a modulatory role for RA that may be important for regulating pericytes during brain vascular development. First, we show that RA mutants (*Rdh10* and *Pdgfbi*^cre^; *dnRAR403*^fl/fl^ mutants) have enhanced vascular β-catenin expression. We believe this is in part due to loss of RA-mediated suppression of *Ctnnb1* in brain endothelial cells. A similar mechanism was recently described in H-1975 cells (a human adenocarcinoma cell line) where RA promotes the activity of GATA6, a transcriptional repressor, to down-regulate *Ctnnb1* expression (Zito et al., 2017). Second, we show that RA has a separate, non-nuclear function, that promotes the phosphorylation and proteasome-mediated degradation of β-catenin in brain endothelial cells via its receptor RARα and PKC activity. RA-RARα regulation of signaling pathways, independent of transcriptional activity, is well-documented. RA-RAR has been shown to regulate ERK signaling (Miloso et al., 2004), insulin-receptor substrate-1 signaling (del Rincon et al., 2004), and CREB activity (Aggarwal et al., 2006). In the brain specifically, membrane associated RARα in hippocampal neurons regulates local translation of mRNA in dendrites and induces new spine formation (Chen and Napoli, 2008; Maghsoodi et al., 2008). Our time course experiments using PLAs indicate that RA-mediated degradation of β-catenin is a faster and, possibly, the more robust mechanism by which RA modulates WNT signaling, especially given the swift interactions (2-4hrs post RA exposure) between RARα, β-catenin and PKCα. Interestingly, RARα appears to interact with PKCα prior to RA stimulus, however, interactions are heightened following RA treatment (4 h). These data support a model in which RARα sits in a complex with PKCα and upon RA stimulation, RARα binds β-catenin and facilitates interactions between β-catenin and PKCα. This leads to the PKC- and RAR-mediated phosphorylation of β-catenin that target it for proteasomal degradation. Interestingly, the reductions in β-catenin and VE-cadherin interactions we observed following RA exposure suggest that RA is capable of degrading β-catenin destined for and/or located at the adherens junctions. Most importantly, we show the interactions between β-catenin with RARα and PKCα are present in the brain vasculature *in vivo*.

In an effort to understand the significance of RA-mediated regulation of endothelial WNT signaling, we investigated WNT-β-catenin regulated BBB properties. Based on data from our mouse mutants with enhanced vascular β-catenin expression and WNT-β-catenin signaling (*Rdh10* and *Pdgfbi*^cre^; *dnRAR403*^fl/fl^ mutants), it is unlikely RA plays a role in regulating the TJ protein Cldn5, a known target of WNT-β-catenin (*Rdh10* mutants; Bonney and Siegenthaler, 2017). We also did not observe overt leakage of fibrinogen indicating that the barrier is intact in the absence of normal RA levels or signaling. Pericyte numbers, however, were increased in the developing brain vasculature suggesting that endothelial RA signaling, functioning upstream of WNT-β-catenin signaling, is involved in regulating pericyte density in the brain vasculature. *Pdgfbi*^cre/+^; *dnRAR403*^fl/fl^ embryos have vascular dysplasia and, in some cases, brain microhemorrhages (Bonney et al., 2016). This suggests that despite the importance of pericytes in the brain, increasing their numbers might impair vascular stability. Possibly, this could be due to enhanced PDGF-B-PDGFrβ signaling causing disruptions in normal pericyte-endothelial cell interactions or crosstalk that is needed for vascular integrity. In support of this, recent investigations in a mouse model of retinopathy showed over-activation of PDGF-B/Pdgfrβ signaling results in excessive pericyte coverage in the retina and promotes the formation of neovascular tufts, which are indicative of endothelial cell over-proliferation. This results in a leaky vascular network that is weak and prone to hemorrhage (Dubrac et al., 2018). Further, expression of a constitutively active form of Pdgfrβ results in impaired brain pericyte differentiation and significantly increased brain capillary diameter (Olson and Soriano, 2011).

Endothelial WNT signaling is uniquely required for brain angiogenesis and establishment of the BBB (Liebner et al., 2008; Stenman et al., 2008; Daneman et al., 2009; Zhou et al., 2014) however, the downstream targets of WNT-β-catenin that mediate its effects on vascular growth and BBB properties are incompletely understood. Endothelial WNT-β-catenin has been previously shown to be sufficient to induce PDGF-B expression and enhance pericyte coverage in brain tumors (Reis et al., 2012) however, to our knowledge, our studies using *Pdgfbi*^cre^; *Ctnnb1*^LOF^ and *Pdgfbi*^cre^; *Ctnnb1*^GOF^ mutants are the first to look at this in the developing brain vasculature. The reduction in pericyte number and *Pdgfb* expression in *Pdgfbi*^cre^; *Ctnnb1*^LOF^ indicate endothelial WNT-β-catenin may be needed to help establish the high number of brain pericytes required for brain vasculature development and maturation. In support of this, comparative endothelial translatome analysis showed *Pdgfb* as well as WNT-β-catenin signaling genes were significantly higher in brain endothelial cells as compared to endothelial cells from whole head, limb, heart, liver, kidney, and lung at E14.5 (Hupe et al., 2017). Based on our data presented here and work by others (Corada et al., 2013; Lee et al., 2015), Sox17 acts downstream of WNT-β-catenin to regulate endothelial *Pdgfb* and pericyte numbers. Moreover, our *in vitro* experiments suggest that Sox17 directly regulates *Pdgfb* expression by binding to the *Pdgfb* locus and promoting its transcription in brain endothelial cells. It is important to note that that deletion of β-catenin or Sox17 in the endothelium results in a fewer brain pericytes, a much milder phenotype than the almost complete lack of brain pericytes observed in *Pdgfb* and *Pdgfr*β knockout mice. This indicates other pathways regulate endothelial PDGF-B and pericyte recruitment in the CNS, likely the same pathways that control PDGF-B in all developing vascular networks. Further, our studies do not address if alterations in the initial recruitment, proliferation or cell death underlie elevated or reduced pericyte numbers in the various mutants. PDGF-B-PDGFrβ signaling is a mitogen, chemoattractant and pro-survival factor for pericytes, suggesting that changes in multiple cellular processes may underlie altered pericyte number in these mutants.

In summary, our model is that, on the one hand, endothelial WNT-Sox17 may provide the additional stimulus of PDGF-B ligand needed to establish high numbers of pericytes that is unique to the CNS vasculature. On the other side, RA balances out WNT-β-catenin signaling and, potentially, PDGF-B expression through Sox17. With the appropriate PDGF-B-PDGFrβ signaling level set by these pathway components, pericytes are able to ‘settle’ in the vasculature and form interactions with and secrete factors onto brain endothelial cells required for vessel stability (Figure 7B).

## Author Contributions

SB and JS designed the research. SB, BD, MW, and JS performed the experiments and acquired the data. SB, BD, and JS analyzed the data. SB and JS wrote and edited the manuscript.

## Conflict of Interest Statement

The authors declare that the research was conducted in the absence of any commercial or financial relationships that could be construed as a potential conflict of interest.

## References

[B1] Aggarwal S. Kim S. W. Cheon K. Tabassam F. H. Yoon J. H. Koo J. S. (2006). Nonclassical action of retinoic acid on the activation of the camp response element-binding protein in normal human bronchial epithelial cells. *Mol. Biol. Cell* 17 566–575. 10.1091/mbc.e05-06-0519 16280361 PMC1356569

[B2] Ashique A. M. May S. R. Kane M. A. Folias A. E. Phamluong K. Choe Y. (2012). Morphological defects in a novel Rdh10 mutant that has reduced retinoic acid biosynthesis and signaling. *Genesis* 50 415–423. 10.1002/dvg.22002 22162152 PMC4118640

[B3] Bauer H. C. Krizbai I. A. Bauer H. Traweger A. (2014). “You shall not pass”-tight junctions of the blood brain barrier. *Front. Neurosci.* 8:392 10.3389/fnins.2014.00392 PMC4253952 25520612

[B4] Bonney S. Harrison-Uy S. Mishra S. Macpherson A. M. Choe Y. Li D. (2016). Diverse functions of retinoic acid in brain vascular development. *J. Neurosci.* 36 7786–7801. 10.1523/JNEUROSCI.3952-15.2016 27445154 PMC4951581

[B5] Bonney S. Siegenthaler J. A. (2017). Differential effects of retinoic acid concentrations in regulating blood-brain barrier properties^∗^. *eNeuro* 4 10.1523/ENEURO.0378-16.2017 28560318 PMC5446490

[B6] Brault V. Moore R. Kutsch S. Ishibashi M. Rowitch D. H. Mcmahon A. P. (2001). Inactivation of the beta-catenin gene by Wnt1-Cre-mediated deletion results in dramatic brain malformation and failure of craniofacial development. *Development* 128 1253–1264. 11262227 10.1242/dev.128.8.1253

[B7] Chanda B. Ditadi A. Iscove N. N. Keller G. (2013). Retinoic acid signaling is essential for embryonic hematopoietic stem cell development. *Cell* 155 215–227. 10.1016/j.cell.2013.08.055 24074870

[B8] Chen N. Napoli J. L. (2008). All-trans-retinoic acid stimulates translation and induces spine formation in hippocampal neurons through a membrane-associated RARαlpha. *FASEB J.* 22 236–245. 10.1096/fj.07-8739com 17712061

[B9] Claxton S. Kostourou V. Jadeja S. Chambon P. Hodivala-Dilke K. Fruttiger M. (2008). Efficient, inducible cre-recombinase activation in vascular endothelium. *Genesis* 46 74–80. 10.1002/dvg.20367 18257043

[B10] Corada M. Orsenigo F. Morini M. F. Pitulescu M. E. Bhat G. Nyqvist D. (2013). Sox17 is indispensable for acquisition and maintenance of arterial identity. *Nat. Commun.* 4:2609. 10.1038/ncomms3609 24153254 PMC3826640

[B11] Damm K. Heyman R. A. Umesono K. Evans R. M. (1993). Functional inhibition of retinoic acid response by dominant negative retinoic acid receptor mutants. *Proc. Natl. Acad. Sci. U.S.A.* 90 2989–2993. 10.1073/pnas.90.7.2989 8096643 PMC46222

[B12] Daneman R. Agalliu D. Zhou L. Kuhnert F. Kuo C. J. Barres B. A. (2009). Wnt/beta-catenin signaling is required for Cns, but not non-Cns, angiogenesis. *Proc. Natl. Acad. Sci. U.S.A.* 106 641–646. 10.1073/pnas.0805165106 19129494 PMC2626756

[B13] del Rincon S. V. Guo Q. Morelli C. Shiu H. Y. Surmacz E. Miller W. H. (2004). Retinoic acid mediates degradation of Irs-1 by the ubiquitin-proteasome pathway, via a Pkc-dependant mechanism. *Oncogene* 23 9269–9279. 10.1038/sj.onc.1208104 15516986

[B14] Dubrac A. Kunzel S. E. Kunzel S. H. Li J. Chandran R. R. Martin K. (2018). Nck-dependent pericyte migration promotes pathological neovascularization in ischemic retinopathy. *Nat Commun.* 9:3463. 10.1038/s41467-018-05926-7 30150707 PMC6110853

[B15] Easwaran V. Pishvaian M. Salimuddin Byers S. (1999). Cross-regulation of beta-catenin-Lef/Tcf and retinoid signaling pathways. *Curr. Biol.* 9 1415–1418. 10.1016/S0960-9822(00)80088-3 10607566

[B16] Engelhardt B. Liebner S. (2014). Novel insights into the development and maintenance of the blood-brain barrier. *Cell Tissue Res.* 355 687–699. 10.1007/s00441-014-1811-2 24590145 PMC3972432

[B17] Gwak J. Cho M. Gong S. J. Won J. Kim D. E. Kim E. Y. (2006). Protein-kinase-C-mediated beta-catenin phosphorylation negatively regulates the Wnt/beta-catenin pathway. *J. Cell Sci.* 119 4702–4709. 10.1242/jcs.03256 17093267

[B18] Hawkins B. T. Davis T. P. (2005). The blood-brain barrier/neurovascular unit in health and disease. *Pharmacol. Rev.* 57 173–185. 10.1124/pr.57.2.4 15914466

[B19] Hupe M. Li M. X. Kneitz S. Davydova D. Yokota C. Kele-Olovsson J. (2017). Gene expression profiles of brain endothelial cells during embryonic development at bulk and single-cell levels. *Sci. Signal.* 10:eaag2476. 10.1126/scisignal.aag2476 28698213

[B20] Lee S. Kim I. K. Ahn J. S. Woo D. C. Kim S. T. Song S. (2015). Deficiency of endothelium-specific transcription factor Sox17 induces intracranial aneurysm. *Circulation* 131 995–1005. 10.1161/CIRCULATIONAHA.114.012568 25596186

[B21] Liebner S. Corada M. Bangsow T. Babbage J. Taddei A. Czupalla C. J. (2008). Wnt/beta-catenin signaling controls development of the blood-brain barrier. *J. Cell Biol.* 183 409–417. 10.1083/jcb.200806024 18955553 PMC2575783

[B22] Lim Y. C. Kang H. J. Kim Y. S. Choi E. C. (2012). All-trans-retinoic acid inhibits growth of head and neck cancer stem cells by suppression of Wnt/beta-catenin pathway. *Eur. J. Cancer* 48 3310–3318. 10.1016/j.ejca.2012.04.013 22640830

[B23] Lohnes D. Mark M. Mendelsohn C. Dolle P. Dierich A. Gorry P. (1994). Function of the retinoic acid receptors (Rars) during development (I). Craniofacial and skeletal abnormalities in Rar double mutants. *Development* 120 2723–2748. 7607067 10.1242/dev.120.10.2723

[B24] Maghsoodi B. Poon M. M. Nam C. I. Aoto J. Ting P. Chen L. (2008). Retinoic acid regulates RARαlpha-mediated control of translation in dendritic RNA granules during homeostatic synaptic plasticity. *Proc. Natl. Acad. Sci. U.S.A.* 105 16015–16020. 10.1073/pnas.0804801105 18840692 PMC2572971

[B25] Messerschmidt D. De Vries W. N. Lorthongpanich C. Balu S. Solter D. Knowles B. B. (2016). beta-catenin-mediated adhesion is required for successful preimplantation mouse embryo development. *Development* 143 1993–1999. 10.1242/dev.133439 27246714

[B26] Miloso M. Villa D. Crimi M. Galbiati S. Donzelli E. Nicolini G. (2004). Retinoic acid-induced neuritogenesis of human neuroblastoma Sh-Sy5Y cells is Erk independent and Pkc dependent. *J. Neurosci. Res.* 75 241–252. 10.1002/jnr.10848 14705145

[B27] Obermeier B. Daneman R. Ransohoff R. M. (2013). Development, maintenance and disruption of the blood-brain barrier. *Nat. Med.* 19 1584–1596. 10.1038/nm.3407 24309662 PMC4080800

[B28] Olson L. E. Soriano P. (2011). Pdgfrβeta signaling regulates mural cell plasticity and inhibits fat development. *Dev. Cell* 20 815–826. 10.1016/j.devcel.2011.04.019 21664579 PMC3121186

[B29] Reis M. Czupalla C. J. Ziegler N. Devraj K. Zinke J. Seidel S. (2012). Endothelial Wnt/beta-catenin signaling inhibits glioma angiogenesis and normalizes tumor blood vessels by inducing Pdgf-B expression. *J. Exp. Med.* 209 1611–1627. 10.1084/jem.20111580 22908324 PMC3428944

[B30] Rosselot C. Spraggon L. Chia I. Batourina E. Riccio P. Lu B. (2010). Non-cell-autonomous retinoid signaling is crucial for renal development. *Development* 137 283–292. 10.1242/dev.040287 20040494 PMC2799161

[B31] Shih S. C. Smith L. E. (2005). Quantitative multi-gene transcriptional profiling using real-time Pcr with a master template. *Exp. Mol. Pathol.* 79 14–22. 10.1016/j.yexmp.2005.03.004 15894312

[B32] Siegenthaler J. Ashique A. Zarbalis K. Patterson K. Hecht J. Kane M. (2009). Retinoic acid from the meninges regulates cortical neuron generation. *Cell* 139 597–609. 10.1016/j.cell.2009.10.004 19879845 PMC2772834

[B33] Siegenthaler J. Choe Y. Patterson K. Hsieh I. Li D. Jaminet S. (2013). Foxc1 is required by pericytes during fetal brain angiogenesis. *Biol. Open* 2 647–659. 10.1242/bio.20135009 23862012 PMC3711032

[B34] Spence J. R. Lange A. W. Lin S. C. Kaestner K. H. Lowy A. M. Kim I. (2009). Sox17 regulates organ lineage segregation of ventral foregut progenitor cells. *Dev. Cell* 17 62–74. 10.1016/j.devcel.2009.05.012 19619492 PMC2734336

[B35] Stenman J. M. Rajagopal J. Carroll T. J. Ishibashi M. Mcmahon J. Mcmahon A. P. (2008). Canonical Wnt signaling regulates organ-specific assembly and differentiation of Cns vasculature. *Science* 322 1247–1250. 10.1126/science.1164594 19023080

[B36] Tsai S. Bartelmez S. Heyman R. Damm K. Evans R. Collins S. J. (1992). A mutated retinoic acid receptor-alpha exhibiting dominant-negative activity alters the lineage development of a multipotent hematopoietic cell line. *Genes Dev.* 6 2258–2269. 10.1101/gad.6.12a.2258 1334022

[B37] Verheyen E. M. Gottardi C. J. (2010). Regulation of Wnt/beta-catenin signaling by protein kinases. *Dev. Dyn.* 239 34–44. 10.1002/dvdy.22019 19623618 PMC3173947

[B38] Ye X. Wang Y. Cahill H. Yu M. Badea T. C. Smallwood P. M. (2009). Norrin, frizzled-4, and Lrp5 signaling in endothelial cells controls a genetic program for retinal vascularization. *Cell* 139 285–298. 10.1016/j.cell.2009.07.047 19837032 PMC2779707

[B39] Zarbalis K. Siegenthaler J. A. Choe Y. May S. R. Peterson A. S. Pleasure S. J. (2007). Cortical dysplasia and skull defects in mice with a Foxc1 allele reveal the role of meningeal differentiation in regulating cortical development. *Proc. Natl. Acad. Sci. U.S.A.* 104 14002–14007. 10.1073/pnas.0702618104 17715063 PMC1955817

[B40] Zhou Y. Wang Y. Tischfield M. Williams J. Smallwood P. M. Rattner A. (2014). Canonical Wnt signaling components in vascular development and barrier formation. *J. Clin. Invest.* 124 3825–3846. 10.1172/JCI76431 25083995 PMC4151216

[B41] Zhu X. Wang W. Zhang X. Bai J. Chen G. Li L. (2015). All-Trans Retinoic Acid-Induced deficiency of the Wnt/beta-catenin pathway enhances hepatic carcinoma stem cell differentiation. *PLoS One* 10:e0143255. 10.1371/journal.pone.0143255 26571119 PMC4646487

[B42] Zito G. Naselli F. Saieva L. Raimondo S. Calabrese G. Guzzardo C. (2017). Retinoic acid affects lung adenocarcinoma growth by inducing differentiation via Gata6 activation and EGFR and WNT inhibition. *Sci. Rep.* 7:4770. 10.1038/s41598-017-05047-z 28684780 PMC5500497

