# Supplementary figures and images for: Retinoic Acid Regulates Endothelial β-catenin Expression and Pericyte Numbers in the Developing Brain Vasculature
Source: Front Cell Neurosci. 2018 Dec 5;12:476. doi: 10.3389/fncel.2018.00476 (PMC6290079; doi:10.3389/fncel.2018.00476)

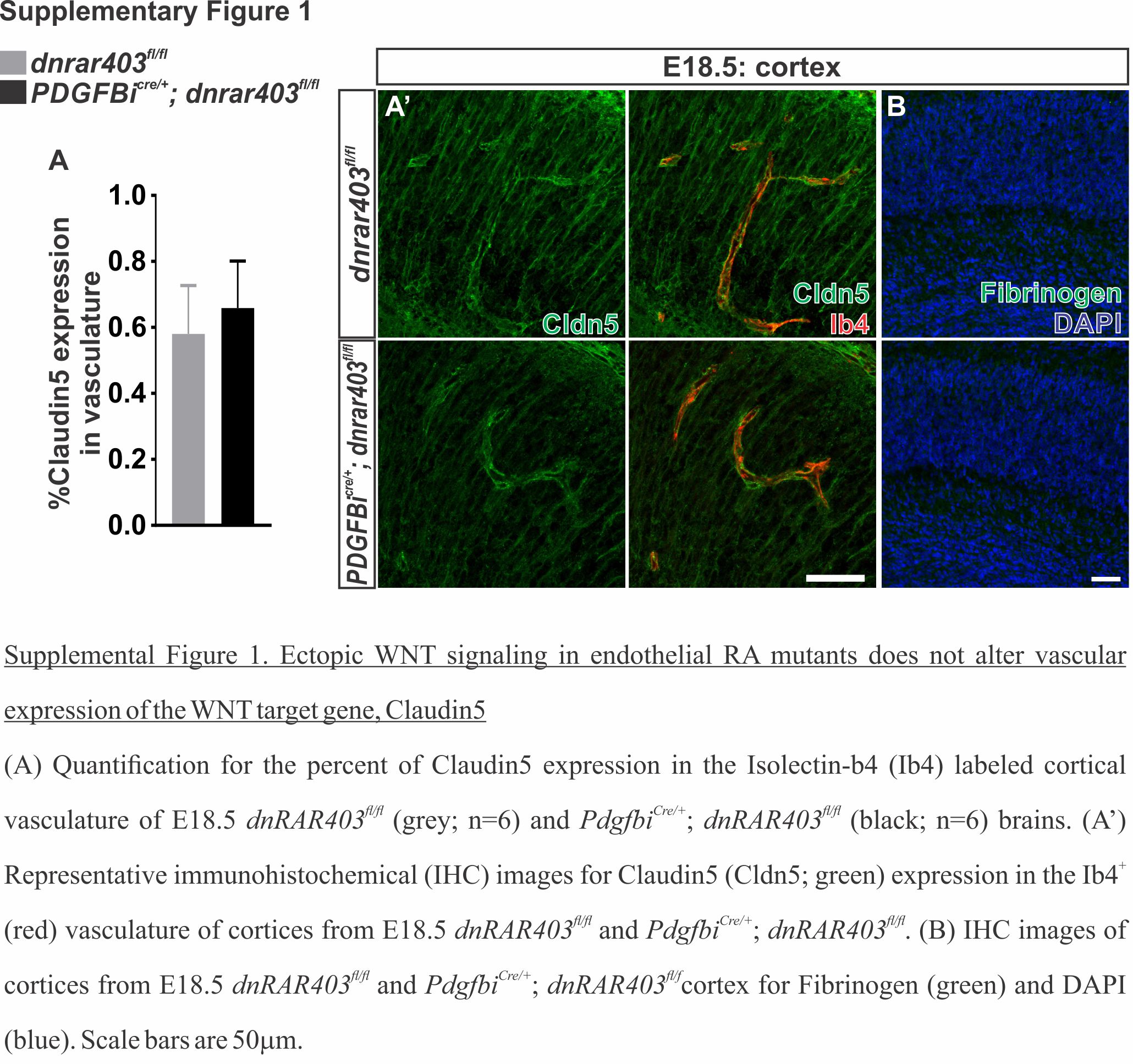

Supplement: Supplementary file 1 [file Image_1.JPEG]
